# Voluntary and Involuntary Control of Attention in Adolescents Born Very Preterm: A Study of Eye Movements

**DOI:** 10.1111/cdev.13310

**Published:** 2019-09-18

**Authors:** E. Sabrina Twilhaar, Artem V. Belopolsky, Jorrit F. de Kieviet, Ruurd M. van Elburg, Jaap Oosterlaan

**Affiliations:** ^1^ Vrije Universiteit Amsterdam; ^2^ Danone Nutricia Research; ^3^ Emma Children's Hospital Amsterdam UMC

## Abstract

Very preterm birth is associated with attention deficits that interfere with academic performance. A better understanding of attention processes is necessary to support very preterm born children. This study examined voluntary and involuntary attentional control in very preterm born adolescents by measuring saccadic eye movements. Additionally, these control processes were related to symptoms of inattention, intelligence, and academic performance. Participants included 47 very preterm and 61 full‐term born 13‐years‐old adolescents. Oculomotor control was assessed using the antisaccade and oculomotor capture paradigm. Very preterm born adolescents showed deficits in antisaccade but not in oculomotor capture performance, indicating impairments in voluntary but not involuntary attentional control. These impairments mediated the relation between very preterm birth and inattention, intelligence, and academic performance.

Attention deficits are prominent in children born very preterm (< 32 weeks’ gestation) and reflected in a two to four times higher risk for attention deficit/hyperactivity disorder in this population (Franz et al., 2018; Johnson & Marlow, 2011). These attention deficits interfere with academic performance (Jaekel, Wolke, & Bartmann, 2013). It is therefore important to further understand specific attention processes in very preterm born children. Of particular interest is the ability to control attention; a core function associated with academic performance and intelligence (McVay & Kane, 2012; Rueda, Checa, & Rothbart, 2010; Unsworth, McMillan, Brewer, & Spillers, 2012). Most studies inferred attention deficits from parent or teacher reports of daily life behavior. Other frequently used measures are reaction time and accuracy on paper‐and‐pencil tasks or computerized experimental paradigms. However, attentional control reflects a highly dynamic interplay between goal‐driven, experience‐driven, and bottom‐up processes (Awh, Belopolsky, & Theeuwes, 2012), which is difficult to measure using manual responses.

Saccadic eye movements provide a direct online measure of attention. There is a tight coupling between attention and saccadic eye movements, such that every eye movement is preceded by a shift of attention (Belopolsky & Theeuwes, 2012; Deubel & Schneider, 1996). The strong relation between attention and eye movements is further validated by substantial overlap in brain networks responsible for attentional shifts with and without eye movements (Corbetta et al., 1998) and by evidence for a functional role of the oculomotor system in directing visual attention (Moore & Fallah, 2001). Oculomotor tasks are typically simple and lack involvement of multiple modalities (e.g., visual, motor, verbal, auditory). Such tasks are particularly suitable for the very preterm population in which impairments are present across domains.

Two experimental paradigms that have been widely used to study the dynamics of attentional control are the *antisaccade* (Hallett, 1978) and *oculomotor capture* (Theeuwes, Kramer, Hahn, & Irwin, 1998) paradigms (Figure 1). In both paradigms, execution of a voluntary saccade toward the target requires goal maintenance and suppression of erroneous reflexive saccades to a salient abruptly appearing stimulus (the onset; Kramer, Gonzalez de Sather, & Cassavaugh, 2005). The most important difference between the two tasks is that in the antisaccade task the onset has to be actively attended to make a correct saccade in the opposite direction, whereas in the oculomotor capture task the onset is completely task‐irrelevant since it does not have to be attended to define the target location (Figure 1). Moreover, in the antisaccade task participants are explicitly instructed not to make a saccade to the onset, whereas no instructions with respect to the onset are provided in the oculomotor capture task. Therefore, erroneous saccades in the oculomotor capture task are assumed to be mostly reflexive in nature, whereas erroneous saccades in the antisaccade task also have an endogenous component (Godijn & Kramer, 2006). This endogenous component is reflected in the key role of working memory for antisaccade execution (Kane, Bleckley, Conway, & Engle, 2001; Kramer et al., 2005). Indeed, working memory capacity has been positively associated with performance in the antisaccade but not in the oculomotor capture task (Kramer et al., 2005).

**Figure 1:**
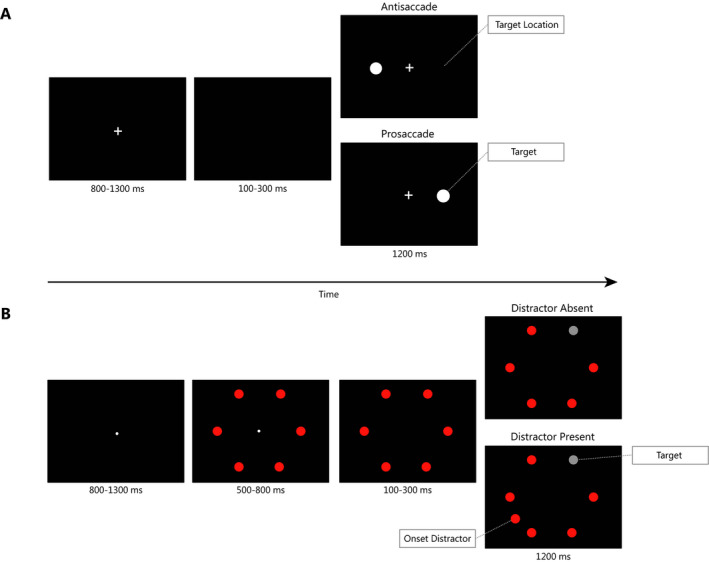
In the antisaccade task (A), participants were required to make a saccade in the direction opposite to the location of the abrupt onset. The onset was physically salient and had to be actively attended to in order to know in which direction to move the eyes. However, the reflexive saccade toward the onset location needed to be suppressed in order to correctly execute an antisaccade. In the oculomotor capture task (B), participants were required to make a saccade toward a target (gray circle), but on half of the trials an onset distractor was presented simultaneously with the target (additional red circle on bottom left). In contrast to the antisaccade task, the onset distractor was completely irrelevant to the task and did not have to be attended in order to plan a saccade to the target. [Color figure can be viewed at wileyonlinelibrary.com]

Only two studies thus far examined oculomotor control in very preterm born children beyond infancy. Newsham, Knox, and Cooke (2007) reported impaired voluntary saccade control as indicated by lower accuracy in the antisaccade task in very preterm than full‐term children aged 8–11 years. In contrast, Loe et al. (2012) found no increase in error rate, but longer saccade latencies on the antisaccade task in very preterm born children aged 9–16 years compared to controls. Moreover, very preterm born children exhibited difficulties maintaining fixation in the presence of peripheral distractors. This study improved on the existing literature by studying attentional control in very preterm and full‐term born adolescents using both the antisaccade and oculomotor capture paradigm. The complementary use of these paradigms enabled us to elucidate to what extent attentional control deficits after very preterm birth result from impaired voluntary or automatic, implicit control processes. Given repeatedly reported working memory deficits in very preterm born children (Clark & Woodward, 2010; Hutchinson et al., 2013), we hypothesized that mainly voluntary control of eye movements is impaired after very preterm birth. Based on the important role of attentional control in academic performance and intelligence (McVay & Kane, 2012; Rueda et al., 2010; Unsworth et al., 2012), we additionally explored whether attentional control processes may serve as underlying mechanisms for symptoms of inattention, cognitive impairment, and academic difficulties after very preterm birth.

## Method

### Participants

Fifty‐five 13‐year‐old children born very preterm (< 32 weeks’ gestation) participated in this study. Participants were recruited from a cohort of 102 very preterm infants who were admitted to the level III neonatal intensive care unit of the Vrije Universiteit Medical Center in Amsterdam between 2001 and 2003 and enrolled in a randomized placebo‐controlled trial on the effects of neonatal enteral glutamine supplementation (Van den Berg, Van Elburg, Twisk, & Fetter, 2004). From the 102 infants originally included in the randomized‐controlled trial in the first month after birth, 13 died in the neonatal period and 1 was excluded because of a chromosomal translocation. This resulted in 88 children eligible for follow‐up. For the current follow‐up study at 13 years of age, 11 children and parents refused to participate, 15 children could not be traced, and 1 had severe disabilities that prevented participation, leaving 61 children that agreed to participate in the assessments. Fifty‐five children and parents completed all measurements and six children and parents only provided questionnaire and academic performance data. Eye‐tracking was unsuccessful for eight very preterm born children, resulting in a sample of 47 children contributing data to this study. Reasons included failed calibration of the eye tracker or failed assessments due to cerebral visual impairment (*n* = 2), fixation instability (*n* = 3), or severe behavioral and neurocognitive disabilities (*n* = 3). Perinatal characteristics of participants and those lost to follow‐up are shown in Table 1. Previous analyses revealed no effects of enteral glutamine supplementation on neurodevelopmental outcomes at age 13 (Twilhaar, de Kieviet, Oosterlaan, & van Elburg, 2018).

**Table 1:** Perinatal Characteristics of Participating and Nonparticipating Very Preterm Born Children in the Study

	Participants (*n* = 47)	Nonparticipants (*n* = 41)	*p*‐Value
Sex, *n* (%) boys	21 (45)	23 (56)	.29^a^
GA, weeks, *M* (*SD*)	29.31 (1.57)	28.95 (2.06)	.35^b^
BW, grams, *M* (*SD*)	1,268.13 (354.17)	1,094.22 (322.64)	.02^b^
SGA^c^, *n* (%)	11 (23)	13 (32)	.38^a^
Caesarean section, *n* (%)	26 (55)	21 (51)	.94^a^
BPD^e^, *n* (%)	12 (26)	15 (37)	.26^a^
IVH grade I/II, *n* (%)	7 (15)	14 (34)	.04^a^
IVH grade III/IV, *n* (%)	0 (0)	3 (7)	.10^d^
PVL, *n* (%)	1 (2)	5 (12)	.09^d^
PDA, *n* (%)	7 (15)	7 (17)	.78^a^
ROP, *n* (%)	2 (4)	6 (15)	.14^d^
NEC, *n* (%)	0 (0)	2 (5)	.21^d^
≥1 serious infection^f^, *n* (%)	28 (60)	28 (68)	.40^a^

Note

GA, gestational age; BW, birth weight; SGA, small for gestational age; BPD, bronchopulmonary dysplasia; IVH, intraventricular hemorrhage; PVL, periventricular leukomalacia; PDA, patent ductus arteriosus; ROP, retinopathy of prematurity; NEC, necrotizing enterocolitis.

a Chi‐square test.

b *t*‐test.

c Birth weight < 10th percentile.

d Fisher's exact test.

e Oxygen requirement at 36 weeks postmenstrual age.

f Sepsis, pneumonia, meningitis, pyelonephritis, or arthritis diagnosed based on a combination of clinical signs and positive culture.

Controls were classmates of very preterm participants or peers from schools located in the same area, born at term (≥ 37 weeks’ gestation), and without developmental, behavioral, or learning disorders. A total of 61 full‐term children participated. Measurements of one child were successful for the antisaccade task, but not for the oculomotor capture task because of difficulties to maintain stable fixation of the eyes on a central fixation point. Only antisaccade data of this participant were used. Demographic characteristics of the very preterm and full‐term born sample are presented in Table 2.

**Table 2:** Sample Characteristics

	Very preterm (*n* = 47)	Full‐term (*n* = 61)	*p*‐Value
Age at assessment, years	13.32 (0.31)	13.27 (0.53)	.51^a^
Sex, *n* (%) boys	21 (45)	27 (44)	.97^b^
Parental education, *n* (%) ≥ bachelor degree or equivalent	28 (60)	38 (62)	.77^b^

a Independent samples *t*‐test.

b Chi‐square test.

### Procedure

The study was executed in accordance with the Declaration of World Medical Association (2013) and approved by the local research ethics committee. All parents and children signed informed consent. Measurements took place in a dimly lit room. Eye movements were registered using a stationary EyeLink 1000 system (SR Research Ltd., Mississauga, ON, Canada) with 1,000 Hz temporal and 0.2° spatial resolution. Saccades were detected using an automatic algorithm that classified an eye movement as a saccade if velocity was > 35°/s and acceleration was > 9,500°/s^2^. A chin rest positioned 70 cm from the screen was used to prevent head movement. Assessments were preceded by a calibration procedure in which participants fixated on nine calibration marks that were randomly presented in a 3 × 3 grid. The procedure was repeated until the maximum validation offset was < 1° and the average validation offset was < 0.5°. Both task order and task conditions were counterbalanced.

### Stimuli

#### Antisaccade Task

Task design is depicted in Figure 1A. The start of each trial required stable fixation on a central fixation cross. After a random duration and a variable gap in which children were instructed to maintain fixation, the onset (white circle, radius: 1°), was presented either to the right or left of the center. Stimulus location was equally distributed and randomized over trials. In the prosaccade condition, children were instructed to look at the stimulus as fast as possible, whereas in the antisaccade condition children were asked to make an eye movement in the direction opposite to the onset as fast as possible. Both conditions included 12 practice and 48 test trials.

#### Oculomotor Capture Task

Stimuli consisted of six red circles (radius: 1.3°) arranged in an imaginary circle (radius: 9.6°), positioned at 0°, 60°, 120°, 180°, 240°, and 300° (Figure 1B). Each trial started with fixation of random duration on a central dot (radius: 0.5°). After a random delay and a variable gap period, one of the circles positioned at 60°, 120°, 240°, or 300° turned gray. This gray circle was the target stimulus. Children were instructed to look at the gray circle as soon as it appeared. In half of the trials an additional red circle, the onset distractor, appeared simultaneously with the target. The position of this onset distractor was always 150° away from the target. Trials were presented in two blocks of 40 trials, preceded by 11 practice trials. Trials with and without onset distractor were evenly and randomly distributed within blocks.

### Outcome Measures

Anticipatory saccades with a latency < 80 ms and saccades with a latency > 600 ms were excluded from further analyses (Fischer, Gezeck, & Hartnegg, 1997). Saccade starting point was required to be within 1.5° from the fixation point. Saccade latency was determined for correct trials and measured as the time (ms) between target onset and initiation of a saccade toward the target. A saccade was classified as correct if the endpoint was within 30° (degrees of arc) from the stimulus’ center in the prosaccade condition and within 30° from the center of the mirrored stimulus location in the antisaccade condition. For the oculomotor capture task, saccades were correct if the endpoint was within 30° from the center of the target. Saccades that landed within 30° from the center of the distractor were classified as erroneous saccades toward the distractor (Godijn & Theeuwes, 2002). Landing error in both tasks describes the deviation (degrees of visual angle) of the saccade landing position from the actual target position. Saccade latency, landing error, and proportion saccades toward the target and distractor were used as measures of task performance. Basic oculomotor function was described by saccade latency, landing error, peak velocity (°/s), and amplitude (°/s) in the control conditions of both tasks (i.e., prosaccade condition and trials without distractor in the oculomotor capture task).

### Statistical Analysis

Group differences in demographic and perinatal characteristics were assessed using independent samples *t*‐test and chi‐square test. For basic oculomotor function, multivariate analyses of variance were performed to test the effect of very preterm birth on saccade latency, landing error, peak velocity, and amplitude in the control condition of each task. For both tasks, mixed‐effects analysis of variance with group (very preterm, full‐term) as between‐subjects factor and task condition (prosaccade, antisaccade and distractor absent, distractor present) as within‐subjects factor was performed to test Group × Condition interactions. Benchmarks for partial η^2^ were .01 (small), .06 (medium), and .14 (large; Cohen, 1988). The proportion of very preterm born children performing > 1 *SD* below the mean of the control group was determined by transforming the difference between conditions in both tasks into *z*‐scores, referenced to the full‐term control group. Mediation analyses were performed to assess whether the relation between very preterm birth and symptoms of inattention, intelligence, and academic performance (task descriptions are provided in the Appendix S1) was mediated by oculomotor control. Analyses were performed using the PROCESS macro for SPSS version 3.0 (Hayes, 2017). Bootstrap 95% confidence intervals for indirect effects were estimated using the percentile method with 5,000 resamples (Preacher & Hayes, 2008). The partially standardized effect size indicates the size of the effect in standard deviation units of the outcome (MacKinnon, 2012).

## Results

**Figure 2:**
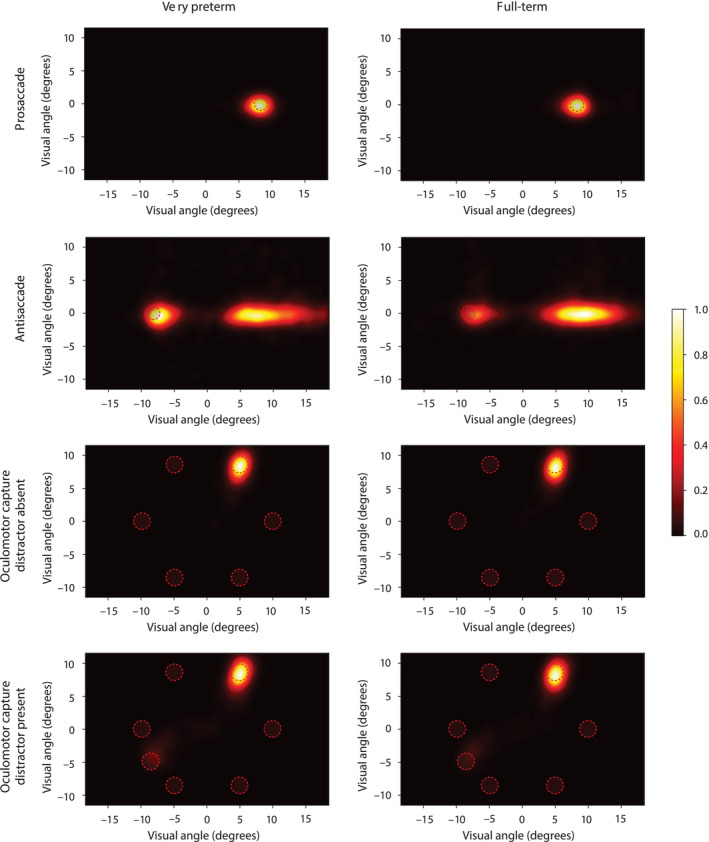
Heatmaps showing a normalized distribution of landing positions of the first saccade in the prosaccade and antisaccade condition and both conditions of the oculomotor capture task for very preterm and full‐term born children. The heatmaps were created by smoothing the landing positions of all participants in each condition with a 2D Gaussian filter with a sigma of 0.6 visual degrees. [Color figure can be viewed at wileyonlinelibrary.com]

Birth weight was higher and prevalence of mild intraventricular hemorrhage was lower in participating compared to nonparticipating very preterm born children (Table 1). Very preterm and full‐term born children did not differ on age, sex, and parental education level (Table 2). Four of the 47 very preterm born children (8.5%) had a diagnosis of attention deficit hyperactivity disorder (ADHD), as reported by parents. The percentage of valid data included in the analysis was 77% for the antisaccade task and 71% for the oculomotor capture task in the very preterm sample and 81% for both tasks in the full‐term sample. Data are visualized in Figure 2, showing heatmaps that provide an overview of saccade landing points of all participants per group and task condition.

### Basic Oculomotor Function

No effects of very preterm birth were found on basic oculomotor function in the prosaccade task, *F*(4, 103) = 0.82, *p *= .51,$\eta_{p}^{2}$ = .03, and trials without onset distractor in the oculomotor capture task, *F*(4, 102) = 0.47, *p *= .76, $\eta_{p}^{2}$ = .02.

### Antisaccade Task

Task condition (prosaccade, antisaccade) significantly affected oculomotor measures. Participants showed longer saccade latency, *F*(1, 106) = 731.01, *p *< .001, $\eta_{p}^{2}$ = .87, increased landing error, *F*(1, 106) = 245.54, *p *< .001, $\eta_{p}^{2}$ = .70, and a smaller proportion of correctly executed saccades, *F*(1, 106) = 223.18, *p *< .001, $\eta_{p}^{2}$ = .68, in the antisaccade relative to the prosaccade condition. No effect was observed on peak velocity, *F*(1, 106) = 2.34, *p *= .13, $\eta_{p}^{2}$ = .02.

The increase in saccade latency and landing error in the antisaccade relative to the prosaccade condition was larger in very preterm born children than controls, as indicated by the significant Condition × Group interaction effects, latency: *F*(1, 106) = 5.81, *p *= .02, $\eta_{p}^{2}$ = .05; landing error: *F*(1, 106) = 7.16, *p *= .01, $\eta_{p}^{2}$ = .06. The Condition × Group interaction effect on peak velocity was not significant, *F*(1, 106) = 2.97, *p *= .09, $\eta_{p}^{2}$ = .03. In addition, very preterm born children showed a smaller proportion of saccades to the target than full‐term controls, *F*(1, 106) = 7.59, *p *= .01, $\eta_{p}^{2}$ = .07. As can be seen from Table 3 and Figure 2, this can be attributed to a larger proportion of erroneous saccades to the onset in the very preterm sample. The proportion of very preterm born children that showed impaired antisaccade performance (i.e., > 1 *SD* from the mean of controls in the direction that indicated performance deficits) was on average, across the different task parameters, 32% (Table 3).

**Table 3:** Antisaccade and Oculomotor Capture Task Performance for Very Preterm (VP) and Full‐Term Born Children and Condition × Group Interaction Effects

	Condition	VP *M* (*SD*)	Full‐term *M* (*SD*)	% VP with impaired performance^a^
Antisaccade				
Latency (ms)	Prosaccade	153.97 (25.72)	161.36 (24.35)	38
	Antisaccade	255.06 (37.79)	245.90 (32.28)	
Landing error (degrees of visual angle)	Prosaccade	1.22 (0.34)	1.21 (0.36)	32
	Antisaccade	4.14 (2.09)	3.28 (1.21)	
Peak velocity (°/s)	Prosaccade	314.75 (56.33)	320.22 (62.27)	23
	Antisaccade	339.03 (114.36)	318.76 (83.43)	
Proportion to target	Prosaccade	1.00 (0.01)	1.00 (0.01)	34
	Antisaccade	0.66 (0.23)	0.76 (0.17)	
Proportion to stimulus	Antisaccade	0.32 (0.22)	0.22 (0.16)	
Oculomotor capture				
Latency (ms)	Distractor absent	219.24 (36.91)	224.72 (32.13)	15
	Distractor present	260.28 (38.03)	257.27 (28.11)	
Landing error (degrees of visual angle)	Distractor absent	1.57 (0.56)	1.63 (0.52)	15
	Distractor present	1.67 (0.57)	1.61 (0.44)	
Peak velocity (°/s)	Distractor absent	346.44 (70.77)	337.23 (59.22)	13
	Distractor present	350.34 (80.72)	339.54 (69.42)	
Proportion to target	Distractor absent	0.98 (0.03)	0.99 (0.02)	26
	Distractor present	0.85 (0.13)	0.88 (0.10)	
Proportion to distractor	Distractor present	0.13 (0.11)	0.10 (0.09)	

a Proportion of children with a standardized difference between conditions (*z*‐score) that deviated > 1 *SD* from the mean of the full‐term sample.

### Oculomotor Capture Task

Presentation of an onset distractor resulted in increased saccade latency, *F*(1, 105) = 213.00, *p *< .001, $\eta_{p}^{2}$ = .67, and a smaller proportion of saccades to the target, *F*(1, 105) = 145.95, *p *< .001, $\eta_{p}^{2}$ = .58, compared to trials without distractor. Landing error, *F*(1, 105) = 1.75, *p *= .19, $\eta_{p}^{2}$ = .02, and peak velocity, *F*(1, 105) = 1.12, *p *= .29, $\eta_{p}^{2}$ = .01, were not affected by the onset.

No significant Condition × Group interaction effects were found for saccade latency, *F*(1, 105) = 2.84, *p *= .10, $\eta_{p}^{2}$ = .03, landing error, *F*(1, 105) = 3.28, *p *= .07, $\eta_{p}^{2}$ = .03, and peak velocity, *F*(1, 105) = 0.07, *p *= .79, $\eta_{p}^{2}$ = .001. Moreover, there was no significant difference between groups in the proportion of saccades to the target when the onset distractor was present, *F*(1, 105) = 2.93, *p *= .09, $\eta_{p}^{2}$ = .03. These findings indicate that in general very preterm and full‐term born children were not differently affected by the presentation of an onset distractor. Based on the different parameters, an average proportion of 17% of the very preterm born children showed impaired performance relative to full‐term controls (Table 3).

### Daily Life Functioning

Mediation analyses revealed that the relation between very preterm birth and parent‐rated symptoms of inattention was mediated by antisaccade performance (i.e., proportion of erroneous saccades toward the onset instead of the opposite target location; Table 4). Also, indirect effects were significant for IQ and academic performance. Partially standardized indirect effects showed that very preterm birth was associated with a 0.16 (95% CI [−0.34, −0.02]), 0.11 (95% CI [−0.26, −0.01]), and 0.11 *SD* (95% CI [−0.23, −0.01]) decrease in inattention score, IQ, and academic performance through its effect on antisaccade performance, respectively.

**Table 4:** Total, Direct, and Indirect Effects of Very Preterm Birth on Parent‐Rated Symptoms of Inattention, IQ, and Academic Performance Through Antisaccade Task Performance

	Point estimate	*SE*	95% CI		*R* ^2^
			Lower	Upper	
Symptoms of inattention^a^					
Total effect	−0.44	0.16	−0.76	−0.11	6%
Direct effect	−0.30	0.16	−0.62	0.02	16%
Indirect effect	−0.14	0.07	−0.30	−0.02	
IQ^b^					
Total effect	−10.86	2.40	−15.61	−6.11	16%
Direct effect	−9.41	2.43	−14.22	−4.59	20%
Indirect effect	−1.45	0.90	−3.56	−0.08	
Academic performance^c^					
Total effect	−0.46	0.16	−0.78	−0.14	7%
Direct effect	−0.35	0.16	−0.68	−0.03	13%
Indirect effect	−0.11	0.06	−0.23	−0.01	

Note

Detailed information on the measurement instruments for symptoms of inattention, IQ, and academic performance is provided in the Appendix S1.

a Measured using the Strengths and Weaknesses of attention deficit hyperactivity disorder Symptoms and Normal Behavior.

b Estimated using the Vocabulary and Block Design subtests of the Wechsler Intelligence Scale for Children, 3rd edition.

c Assessed using a Dutch pupil monitoring system developed by the National Institute for Educational Measurement.

Within the very preterm sample, the proportion of saccades toward the onset in the antisaccade task was significantly associated with parent‐reported symptoms of inattention (*r *= −.37, *p *= .01) and IQ (*r *= −.32, *p *= .03). The relation with academic performance was not significant (*r *= −.27, *p *= .07).

## Discussion

This study aimed to elucidate attentional control deficits after very preterm birth by studying oculomotor control in very preterm and full‐term born adolescents. The use of two well‐validated paradigms, the antisaccade and oculomotor capture task, enabled to what extent attentional control deficits after very preterm birth result from impaired voluntary control processes or from failure to suppress automatic processes. Very preterm born adolescents showed difficulties intentionally inhibiting reflexive saccades toward task‐relevant information, but not with automatically inhibiting reflexive saccades toward task‐irrelevant information. This indicates deficits in voluntary attentional control. Furthermore, voluntary control of saccades mediated the association between very preterm birth and symptoms of inattention, intelligence, and academic performance. Also, among very preterm born children, poorer voluntary control of saccades was associated with more symptoms of inattention and lower intelligence. The association with academic performance was of similar strength, but did not reach the threshold for statistical significance.

Successful antisaccade execution involves maintenance of a complex set of task goals in working memory and suppression of a reflexive saccade to the stimulus. In order to suppress a reflexive saccade in antisaccade trials, saccade neurons in the frontal eye field and superior colliculus are to be inhibited (Munoz & Everling, 2004). This inhibition occurs both before and in response to stimulus presentation. Projections from neurons in the dorsolateral prefrontal cortex to the superior colliculus play a crucial role in the selective inhibition of neural activity in the superior colliculus and the subsequent suppression of reflexive prosaccades, thereby facilitating the execution of an antisaccade (Johnston & Everling, 2006; Meeter, Van der Stigchel, & Theeuwes, 2010). While the suppression of reflexive saccades is required in both the antisaccade and oculomotor capture task, the maintenance of a complex set of goals is not required in the oculomotor capture task. The impairments in voluntary as opposed to involuntary control of saccades therefore suggest that very preterm born adolescents have particularly difficulties maintaining such a complex set of goals. In addition, antisaccade execution requires maintenance of a representation of the stimulus location. The increased landing error for antisaccades in very preterm adolescents may be explained by difficulties maintaining a representation of the stimulus location. This maintenance of task goals and stimulus location primarily relies on working memory capacity, with a crucial role of the dorsolateral prefrontal cortex (Barbey, Koenigs, & Grafman, 2013). Previous studies established the association between antisaccade performance and working memory (Kane et al., 2001; Kramer et al., 2005). Kramer et al. (2005) found that antisaccade performance improves until age 15, whereas oculomotor capture performance is stable from childhood to young adulthood. Improvement of antisaccade performance coincides with the development of working memory that continues into adolescence (Crone, Wendelken, Donohue, van Leijenhorst, & Bunge, 2006; Luciana, Conklin, Hooper, & Yarger, 2005). Moreover, Eenshuistra, Ridderinkhof, Weidema, and Van der Molen (2007) showed, consistent with findings from Roberts, Hager, and Heron (1994), that the poorer suppression of reflexive saccades in the antisaccade task of children relative to adults was explained by their smaller working memory capacity rather than inefficient inhibitory control mechanisms. As deficits in working memory have been frequently reported in very preterm born children (Clark & Woodward, 2010; Hutchinson et al., 2013), we suggest that working memory capacity plays an important role in the impaired voluntary control of eye movements.

Antisaccade performance involves activation of a fronto‐subcortical‐parietal network (Jamadar, Fielding, & Egan, 2013). Hwang, Velanova, and Luna (2010) showed that better oculomotor control in adults compared to adolescents and children was associated with enhanced top‐down connectivity between frontal and parietal and subcortical regions. Children showed a characteristic profile of short‐range connections within the parietal cortex. During adolescence, these short‐range connections weakened, whereas connections from frontal to downstream regions strengthened, which was associated with improved oculomotor control (Hwang et al., 2010). Preterm birth is associated with alterations in white matter microstructure in adolescence and adulthood (De Kieviet, Zoetebier, Van Elburg, Vermeulen, & Oosterlaan, 2012; Eikenes, Løhaugen, Brubakk, Skranes, & Håberg, 2011), which are associated with impaired executive functions (Vollmer et al., 2017). These alterations may be responsible for weakened control of frontal regions over downstream regions and may result in impaired suppression of reflexive saccades and execution of voluntary saccades.

In line with observations from research and clinical practice, performance in the very preterm sample was marked by heterogeneity. Further exploration of this heterogeneity revealed that the majority of very preterm born children performed below the average of the full‐term sample, although a significant proportion performed within 1 *SD* from the mean. However, another substantial part of the very preterm born children, about one third, showed performance that deviated more than 1 *SD* from the average performance of the full‐term sample. This indicates that impairments in voluntary control of eye movements were not limited to a small subgroup of very preterm born children, for example, those children with a diagnosis of ADHD in our sample (8.5%). This also emphasizes the importance for future research to not only observe heterogeneity, but also try to identify factors that may explain the large differences among very preterm born children in neurodevelopmental outcomes in general and in attentional control in particular.

The present findings have important clinical implications. First, very preterm birth was associated with impairments in the voluntary control of saccades as opposed to the suppression of task‐irrelevant information. Very preterm born children may therefore benefit from strategies that support the active maintenance of complex sets of goals instead of merely reducing task‐irrelevant distractors in the environment to improve daily life functioning. One widely studied strategy to improve goal maintenance is working memory training. However, a critical review of the literature does not provide evidence for its efficacy, that is, transfer of effects to cognitive domains other than the trained task (Shipstead, Redick, & Engle, 2012). Currently, there are no interventions available that may improve attentional control or broader neurocognitive outcomes in very preterm born children and more research is necessary in this respect. A second implication of the current findings relates to the fact that impaired voluntary saccade control has been associated with psychopathology and neurocognitive dysfunction (Hutton & Ettinger, 2006; Sweeney, Takarae, Macmillan, Luna, & Minshew, 2004). Also in this study, impairments in voluntary control of saccades explained the increased symptoms of inattention and poorer intelligence and academic performance in very preterm born adolescents compared to full‐term peers. Moreover, heterogeneity among very preterm born children with respect to these outcomes was explained by antisaccade performance. This implicates the possible usefulness of the antisaccade paradigm for early identification of very preterm born children at risk for impairments in a broader range of cognitive and behavioral domains. However, longitudinal studies are required to establish the relation between early oculomotor control and later developmental outcomes in this population.

While the antisaccade paradigm has generally been used in children aged six and older (Klein & Foerster, 2001), several oculomotor paradigms have successfully been applied to study visual attention in infancy (Colombo, 2001). A recent eye movement study by Downes, Kelly, Day, Marlow, and de Haan (2018) showed deficits in attentional control in preterm compared to full‐term born infants at 12 months of corrected age. Whether these deficits are predictive of impairments that are commonly observed in childhood after very preterm birth is yet unknown, but individual differences in attentional control processes in full‐term born infants have been found to predict childhood executive function, effortful control, and hyperactivity–impulsivity (Papageorgiou et al., 2014; Rose, Feldman, & Jankowski, 2012). Eye‐tracking could therefore be a useful method to find objective markers of later neurocognitive impairments after very preterm birth in the infancy period, before children go to school or are able to perform complex neurocognitive test and when deficits may be too subtle to detect through questionnaires or behavioral assessments. This may facilitate the monitoring and provision of early intervention in very preterm born children that have an increased risk for poor outcomes.

The results of this study need to be interpreted in light of the following limitations. First, this study reports on a relatively small sample of very preterm born children in which we found evidence for selective loss to follow‐up and high parental education levels. Consequently, deficits reported in this study likely underestimate deficits in the population. Furthermore, for eight children in the very preterm sample against one child in the full‐term sample measurements were (partly) unsuccessful due to specific or nonspecific eye problems. This is a relevant observation, but may also have resulted in further attrition of a specific group of children since visual impairments in very preterm born children are associated with lower gestational age (Blencowe, Lawn, Vazquez, Fielder, & Gilbert, 2013) and poorer neurodevelopmental outcomes (Schmidt, Davis, Asztalos, Solimano, & Roberts, 2014).

In conclusion, very preterm born adolescents showed deficits in antisaccade performance, but not oculomotor capture performance. This indicates impaired voluntary control of eye movement that involves maintenance of a complex set of goals rather than merely suppressing distracting information. Impaired antisaccade performance played a mediating role in the association between very preterm birth and functional outcomes and was associated with poorer functional outcomes within the very preterm sample. These findings contribute to our understanding of the specific neurocognitive processes that are affected by very preterm birth and that underlie problems in domains of general functioning. This knowledge is necessary for monitoring and support of very preterm born children. The well‐studied antisaccade and oculomotor capture paradigms are recommended for future studies in this population, because it allows a relatively direct measurement of covert attentional processes that is not confounded by impaired motor function or speech.

## Supporting information


**Appendix S1.** Description of Secondary Outcome Measures
