# Supplementary material for: Voluntary and Involuntary Control of Attention in Adolescents Born Very Preterm: A Study of Eye Movements
Source: Child Dev. 2019 Sep 18;91(4):1272–83. doi: 10.1111/cdev.13310 (PMC7497183; doi:10.1111/cdev.13310)
Supplement: Supplementary file 1 — Appendix S1. Description of Secondary Outcome Measures [file CDEV-91-1272-s001.docx]

**Description of secondary outcome measures**

*Symptoms of inattention*

The Strengths and Weaknesses of ADHD Symptoms and Normal Behavior (Swanson et al., 2012) was used as a parent-rated measure of inattention in daily life. This questionnaire was developed to capture variance at the symptomatic as well as the adaptive ends of the attention dimension. Behavior was rated on a seven-point Likert scale, ranging from -3 (far below average) to +3 (far above average), anchored to average behavior. The inattention subscale score was calculated by the sum of the item scores divided by the number of items (Swanson et al., 2012).

*Intelligence*

Intelligence was assessed using the Vocabulary and Block Design subtests of the Dutch version of the Wechsler Intelligence Scale for Children, 3^rd^ edition (Wechsler, 1991) to estimate the full-scale intelligence quotient (IQ). This short form has excellent reliability (*r* = 0.91) and correlates strongly (*r* > 0.90) with full-scale IQ (Sattler, 2008).

*Academic performance*

The Dutch pupil monitoring system developed by the National Institute for Educational Measurement (Gillijns & Verhoeven, 1992) includes standardized tests to assess academic performance during primary school (Janssen, Verhelst, Engelen, & Scheltens, 2010; Mols & Kamphuis, 2010; Weekers, Groenen, Kleintjes, & Feenstra, 2011). For the current study, the most recent test scores (i.e. grade six test scores for most participants) for arithmetic, reading comprehension, and spelling were used. The tests are based on item response theory. Reliability coefficients for arithmetic, reading comprehension, and spelling are higher than 0.90, 0.83, and 0.89 respectively (Janssen, Verhelst, Engelen, & Scheltens, 2010; Weekers, Groenen, Kleintjes, & Feenstra, 2011; Mols & Kamphuis, 2010). Test performance is reflected in a standardized ability score on a unidimensional scale. To facilitate interpretation, ability scores were transformed into *z*-scores and averaged into one academic performance variable.

**References**

Gillijns, P., & Verhoeven, L. (1992). Het CITO leerlingvolgsysteem: Met het oog op de praktijk

[The CITO pupil monitoring system: Focus on practice]. *Pedagogische Studiën*, *69*, 291-296.

Janssen, J., Verhelst, N., Engelen, R., & Scheltens, F. (2010). *Wetenschappelijke*

*verantwoording van de toetsen LOVS rekenen-wiskunde voor groep 3 tot en met 8*

*[Scientific justification of the mathematics test for grade 1 until grade 6]*. Arnhem: Cito.

Mols, A., & Kamphuis, F. (2010). *Wetenschappelijke verantwoording van de toetsen spelling*

*niet-werkwoorden voor groep 7 en 8 [Scientific justification of the spelling test for grade*

*5 and 6].* Arnhem: Cito.

Sattler, J.M. (2008). *Assessment of children: cognitive foundations.* San Diego: J.M. Sattler Inc.

Swanson, J.M., Schuck, S., Porter, M.M., . . . et al. (2012). Categorical and dimensional

definitions and evaluations of symptoms of ADHD: History of the SNAP and the SWAN rating scales. *International Journal of Educational and Psychological Assessment, 10,* 51-70.

Wechsler, D. (1991). *Wechsler Intelligence Scale for Children, third edition.* San Antonio:

Psychological Corporation.

Weekers, A., Groenen, I., Kleintjes, F., & Feenstra, H. (2011). *Wetenschappelijke*

*verantwoording papieren toetsen begrijpend lezen voor groep 7 en 8 [Scientific justification of the reading comprehension test for grade 5 and 6].* Arnhem: Cito.
